# Effect of an education program on improving knowledge of schizophrenia among parents of junior and senior high school students in Japan

**DOI:** 10.1186/1471-2458-11-323

**Published:** 2011-05-17

**Authors:** Hatsumi Yoshii, Yuichiro Watanabe, Hideaki Kitamura, Jun Chen, Kouhei Akazawa

**Affiliations:** 1 Department of Medical Informatics and Statistics, Niigata University Graduate, School of Medicine 1-754 Asahimachi, Chuo-ku, Niigata, Japan 951-8520; 2 Department of Psychiatry, Niigata University Graduate School of Medicine 1-754 Asahimachi, Chuo-ku, Niigata, Japan 951-8520

## Abstract

**Background:**

Early detection and intervention in schizophrenia are important in improving quality of life after treatment and are major issues in psychiatric care. Therefore, it is necessary to increase knowledge of schizophrenia among the general public. Among parents of junior and senior high school students in Japan, we compared rates of correct answers for items on knowledge of schizophrenia and ability to discriminate this psychosis from other disorders on questionnaires given before and after viewing a web-based education program.

**Methods:**

Questionnaires were distributed to 2,690 parents. The program was developed to help parents obtain a basic understanding of schizophrenia and to emphasize the necessity of early detection.

**Results:**

Before the program, the rate of correct answers was 77% for items concerning basic knowledge of schizophrenia, 47% for "discrimination of schizophrenia symptoms," and 30% for "discrimination of prodromal symptoms." The program resulted in an improvement in basic knowledge of schizophrenia, discrimination of schizophrenia symptoms, and discrimination of prodromal symptoms (*P *< 0.001 for all).

**Conclusions:**

Our web-based education program was useful in helping parents acquire a basic knowledge of schizophrenia and discriminate correctly the symptoms of schizophrenia.

## Background

Delayed detection of schizophrenia and the resulting prolongation of the duration of untreated psychosis (DUP) are viewed as serious issues in the mental health field [1-3]. The median DUP has been estimated at between 1 and 2 years [4,5], and a prolonged DUP has been associated with delayed remission, increased risk of suicide, substance abuse, and an unfavourable prognosis [6-8].

Studies have revealed a number of important factors related to delayed detection of schizophrenia [9-13]. One is lack of basic knowledge of the symptoms of the disease [9-12]. Those without an understanding of the symptoms of schizophrenia may miss the onset of the illness in people close to them.

Providing accurate information on schizophrenia is essential in preventing delays in diagnosis. In particular, parents of adolescents need to have sufficient knowledge of the disorder so they can identify an initial episode in their children. The onset of schizophrenia is typically between ages 15 and 25 years in males and between ages 25 and 35 years in females [4]. However, individuals are likely to experience their first psychotic symptoms before age 20 [14]. If parents are able to identify the symptoms of schizophrenia at an early stage, they are more likely to seek appropriate medical care for their children [1,7].

In recent years, several sophisticated educational programs have been developed to provide basic information, under appropriate conditions, on schizophrenia and its associated behaviours to various populations [15-17]. However, there have been no studies regarding the effectiveness of such programs among parents of junior and senior high school students. In this study, we use a multifaceted questionnaire to evaluate knowledge of schizophrenia. Furthermore, we investigate the effectiveness of a web-based education program that aimed to improve understanding of schizophrenia among 2,690 Japanese parents of adolescents.

## Methods

### Setting and participants

The participants were extracted from a large database of 1,370,000 candidates that is administered by a Japanese private company specializing in questionnaire research. First, we extracted 44,000 parents of junior and senior high school students, all registered in the database. Second, gender and region were used as variables for stratified random sampling. Finally, 5,000 final candidates were extracted. Thus, the gender and region distributions of our sample were almost identical to those of the Japanese general population. Of the 5,000 candidates, 1,410 parents of junior high school students and 1,280 parents of senior high school students participated in this survey (the "first survey"). All respondents completed a questionnaire on an internet website that was newly developed for this study by the survey company. To evaluate the reliability of the first survey, we performed another survey of basic knowledge of schizophrenia and ability to discriminate it from other disorders in the same manner as the first survey. We used the same questionnaire for a different sample of 735 individuals who had not viewed our web-based education program (the "second survey"). All respondents were Japanese adults living in Japan. The study was approved by the medical ethics committee of Niigata University.

### Questionnaire

The questionnaire used in the present study consisted of 3 sections. Section 1 collected demographic information on respondents including age, occupation, education attainment, and family structure (Table 1). Section 2 comprised 14 true/false items on basic knowledge of schizophrenia that were developed for this study. The items addressed the causes, symptoms, incidence rate, and treatment of schizophrenia (Table 2). Section 3 examined the ability to discriminate schizophrenia symptoms from those of other disorders (Appendix I). It was based on the *Diagnostic and Statistical Manual of Mental Disorders, Fourth Edition, Text Revision *(DSM-IV-TR) criteria for schizophrenia and the PRIME Screen [18]. The PRIME Screen is an assessment instrument used to identify, at an early stage, subjects with prodromal symptoms. The questionnaire comprised 4 items on schizophrenia symptoms, 4 on prodromal symptoms, 4 on other mental illnesses, 2 on physical disorders, and 4 items on individuals who display unusual or antisocial behaviour. The respondents answered Sections 2 and 3 twice, before and after the education program (referred to as the "before" questionnaire and "after" questionnaire, respectively).

**Table 1:** Demographic characteristics of respondents (*n *= 2,690)

Characteristic	n	%	Demographic characteristics of parents in Japan (%)*
**Age (years)**			
30-39	221	8.2	31.2
40-49	1,904	70.8	37.6
50-59	548	20.4	14.0
60-69	17	0.6	5.5
Others	0	0	11.7
**Gender**			
Male	1,381	51.3	
Female	1,309	48.7	
**Education**			
Junior high school	25	0.9	9.7
High school	766	28.5	55.0
Vocational school	349	13.0	8.25
Junior college	385	14.3	8.25
University	1,063	39.5	17.15
Graduate school	96	3.6	1.1
Others	6	0.2	0.55
**Family structure**			
2 parents	2,092	77.8	69.0
1 parent	89	3.3	7.0
3 generations	466	17.3	19.8
Others	43	1.6	4.2
**Family income**			
(US dollars)			
< 11,000	41	1.5	1.5
11,000-32,000	196	7.3	12.8
32,000-53,000	502	18.7	21.8
53,000-110,000	1,465	54.5	47.6
> 110,000	486	18.0	16.3
**Proximity to person with schizophrenia**			
Yes	88	3.3	
No	2,602	96.7	
**Occupation**			
Agriculture, forestry and fisheries	11	0.4	2.6
Production labour service	631	23.5	18.8
Transportation and communication	141	5.2	14.1
Sales and marketing	349	13.0	32.6
Service industry	340	12.6	9.4
Professionals	482	17.9	21.1
Others	160	6.0	1.2
Unemployed	576	21.4	0.2
**Participation in welfare activities for people with mental illness**			
Yes	222	8.3	
No	2468	91.7	

* The Cabinet Office: http://www8.cao.go.jp/youth/kenkyu.htm

Ministry of Health, Labour and Welfare:http://www.mhlw.go.jp/toukei/list/20-21.html

**Table 2:** Rates (%) of correct answers for the 14 items on basic knowledge of schizophrenia

Item	Correct answer	"before" (*n *= 2,690)	"after" (*n *= 2,465)	***P***
1. The incidence of schizophrenia is 1 out of 100 individuals.	True	9.9	33.8	< 0.001
2. Junior and senior high school students do not develop schizophrenia.	False	99.1	98.4	< 0.05
3. Schizophrenia is not a mental illness.	False	93.8	90.7	< 0.001
4. Differences in child-rearing methods are not responsible for the onset of the illness.	True	20.5	35.5	< 0.001
5. A person with a mild disposition will not necessarily develop schizophrenia.	True	29.2	34.6	< 0.001
6. Schizophrenia has been proven to be a genetic disease.	False	97.7	97.7	NS
7. A person with schizophrenia may have little will power.	False	97.4	96.8	NS
8. There is no effective medication for schizophrenia.	False	94.1	95.3	< 0.05
9. It is not possible to recover from schizophrenia, even with treatment.	False	97.8	98.1	NS
10. Auditory hallucinations and delusions are not symptoms of schizophrenia.	False	97.9	97.9	NS
11. Poor concentration and fatigue are not symptoms of schizophrenia.	False	98.0	98.1	NS
12. Social withdrawal is not a symptom of schizophrenia.	False	92.8	95.3	< 0.001
13. The appearance and duration of symptoms can vary among individuals.	True	65.7	61.7	< 0.05
14. All the above items are incorrect.	False	83.8	85.8	< 0.05
Total score		77.0	80.0	< 0.001

### Web-based education program

After the "before" questionnaire survey was conducted, all respondents were invited to view a web-based education program that had been developed to increase understanding of schizophrenia, after which the "after" questionnaire survey was conducted using the same questionnaire for the same respondents. The content of the education program consisted of 10 units: (1) characteristics of schizophrenia, (2) causes of schizophrenia, (3) symptoms of schizophrenia, (4) classification of schizophrenia, (5) course of the disease and its characteristic clinical features, (6) treatment of schizophrenia, (7) prognosis, (8) how to prevent progression and exacerbation of the disorder, (9) signs of progression, and (10) consultation facilities.

The slide show included 12 slides with narration and required 13 minutes to complete. The media were delivered via the same internet website that was used for the questionnaire survey. The effectiveness of the education program was evaluated by the same respondents 1 week later.

### Statistical analysis

All analyses were performed using the Statistical Package for the Social Sciences (SPSS) version 16.0. McNemar's test was used to compare the proportion of specific answers in paired data, that is, the results of the "before" and "after" questionnaires for each respondent. An unpaired t-test and analysis of variance (ANOVA) were used to compare the effect of the web-based education program on basic knowledge of schizophrenia and the ability to discriminate it from other disorders among several groups of demographic characteristics. The endpoint was the correct answer score, calculated by dividing the sum of the differences between the answers (correct = 1 and incorrect = 0) for the "after" questionnaire and those for the "before" questionnaire for all respondents by the number of items (i.e. 14). All statistical tests were 2-tailed, and statistical significance was defined as *P *< 0.05.

## Results

### Characteristics of respondents

In total, 2,690 of the 5,000 individuals completed the "before" questionnaire, and 2,465 completed the "after" questionnaire after viewing the education program. Mean age ± standard deviation at questionnaire completion was 45.9 ± 4.7 years, and approximately 71% of the respondents were in their 40 s (Table 1). The 2,690 respondents included 1,381 (51.3%) men and 1,309 (48.7%) women. Regarding educational attainment, 1,159 (43.1%) respondents had completed university or graduate school. A total of 2,092 (77.8%) respondents reported being part of a 2-parent household. The majority of respondents (54.5%) reported a family income of 53,000 to 110,000 US dollars. Eighty-eight (3.3%) reported living close to someone with schizophrenia. For reference, selected corresponding demographic characteristics of parents in the Japanese general population are shown in Table 1.

### Basic knowledge of schizophrenia

Table 2 shows the rate of correct answers on the questionnaires given before and after the education program for the 14 items on basic knowledge of schizophrenia. In the "before" questionnaire, all items except numbers 1, 4, 5, and 13 had a rate of correct answers higher than 90%. The item with the lowest rate (9.9%) of correct answers was number 1, which asked about the incidence of schizophrenia. Other items with a low rate of correct answers were numbers 4 and 5 (20.5% and 29.2%, respectively). These findings revealed that respondents were unaware that child-rearing methods and personality are not responsible for the onset of schizophrenia. The identical questionnaire was administered after the respondents had viewed the education program on the same website. For six items--1, 4, 5, 8, 12, and 14--the rates of correct answers on the "after" questionnaire were significantly higher than those on the "before" questionnaire. The extent of the change in correct answer rates after viewing the web-based education program was analysed among several categories of demographic variables using the unpaired t-test and ANOVA. Significant differences in correct answer scores were associated with age, gender, education, and participation in welfare activities for people with mental illness (Table 3). The results of the second survey, which was administered to evaluate the reliability of the results of the first survey, are shown in Appendix II (Tables 5 and 6).

**Table 3:** Demographic characteristics and correct answer scores for basic knowledge of schizophrenia and discrimination between other disorders

	Basic knowledge of schizophrenia		Discrimination between disorders	
	
	Mean ± SD	P**	Mean ± SD	P**
**Age (years)**		0.019		0.050
30-39	0.51 ± 2.03		1.48 ± 4.36	
40-49	0.36 ± 1.95		0.71 ± 4.32	
50-59	0.66 ± 1.96		1.13 ± 4.45	
60-69	0.81 ± 2.20		0.81 ± 5.23	
**Gender**		0.001		0.156
Male	0.57 ± 1.95		0.97 ± 4.28	
Female	0.28 ± 1.97		0.72 ± 4.45	
**Education**		0.021		0.001
Junior high school	1.81 ± 1.40		2.33 ± 2.89	
High school	0.45 ± 1.94		1.34 ± 4.26	
Vocational school	0.41 ± 1.95		1.14 ± 4.45	
Junior college	0.23 ± 1.98		0.52 ± 4.44	
University	0.48 ± 1.98		0.64 ± 4.37	
Graduate school	0.49 ± 2.03		-0.26 ± 4.26	
Others	1.00 ± 1.00		0.00 ± 4.18	
**Family structure**		0.603		0.019
2 parents	0.44 ± 1.95		0.82 ± 4.35	
1 parent	0.16 ± 1.87		0.40 ± 5.04	
3 generations	0.50 ± 2.00		1.26 ± 4.32	
Others	0.40 ± 2.25		-0.69 ± 3.48	
**Family income (US dollars)**		0.154		0.003
< 11,000	1.00 ± 2.01		0.46 ± 4.88	
11,000-32,000	0.67 ± 1.79		1.72 ± 4.80	
32,000-53,000	0.48 ± 2.01		1.28 ± 4.28	
53,000-110,000	0.39 ± 1.98		0.75 ± 4.23	
> 110,000	0.41 ± 1.93		0.45 ± 4.51	
**Proximity to person with schizophrenia**		0.056		0.004
Yes	0.04 ± 2.03		-0.48 ± 5.01	
No	0.45 ± 1.96		0.91 ± 4.33	
**Occupation**		0.167		0.001
Agriculture, forestry and fisheries	1.00 ± 1.63		3.30 ± 3.77	
Production labour service	0.6 ± 1.96		0.96 ± 4.20	
Transportation and communication	0.66 ± 2.14		1.16 ± 4.22	
Sales and marketing	0.48 ± 2.02		0.94 ± 4.44	
Service industry	0.41 ± 1.92		1.34 ± 4.57	
Professionals	0.28 ± 1.92		0.07 ± 4.41	
Others	0.56 ± 1.88		1.23 ± 3.75	
Unemployed	0.27 ± 1.97		0.84 ± 4.42	
**Participation in welfare activities for people with mental illness**		0.001		0.193
Yes	-0.14 ± 2.02		0.48 ± 4.60	
No	0.49 ± 1.95		0.89 ± 4.33	

* Correct answer scores were calculated by dividing the sum of the differences between the answers (correct = 1 and incorrect = 0) on the "after" questionnaire with those on the "before" questionnaire for all respondents by the number of items.

** Student's or Welch's t-test, One-way analysis of variance.

### Rates of discrimination between disorders

We evaluated the rates of correct answers for the questionnaire section on discriminating between schizophrenia symptoms and other disorders (Table 4). The lowest rates of correct answers in the "before" questionnaire were for discrimination of prodromal symptoms (30%) and discrimination of schizophrenia symptoms (47%), indicating that respondents lacked knowledge of the symptoms of schizophrenia and its prodromal phase. For discrimination of other mental illnesses, physical disorders, and individuals who display unusual or antisocial behaviour, the respective rates of correct answers were 65%, 93%, and 93%. However, the percentage of correct answers on the "after" questionnaire improved to 61% for both discrimination of prodromal symptoms and discrimination of schizophrenia symptoms. McNemar's test revealed that these increases were statistically significant. The unpaired t-test and ANOVA indicated that five variables--education, family structure, family income, proximity to a person with schizophrenia, and occupation--were associated with significant differences in the means of correct answer scores (Table 3). The results of the second survey are shown in Appendix III (Table 7).

**Table 4:** Rates (%) of correct answers for 5 items on discrimination between disorders

Item	"before"	"after"	P*
Discrimination of schizophrenia symptoms	47	61	< 0.001
Discrimination of prodromal symptoms	30	61	< 0.001
Discrimination of other mental illnesses	65	61	< 0.01
Discrimination of physical disorders	93	89	< 0.001
Discrimination of individuals who display unusual or antisocial behaviour	93	88	< 0.001

*McNemar's test

## Discussion

We developed an experimental web-based education program to aid the parents of adolescents in quickly acquiring a broad knowledge of schizophrenia. In particular, we focused on knowledge regarding detection of the prodromal stage and symptoms of the disorder. Parents were able to browse the content of the program on an internet website at a time of their convenience and learn about schizophrenia, including its characteristics, causes, symptoms, classification, treatments, and prognosis. Our education program has two important features. The first is that the information was condensed so it could be presented on only 12 slides viewed over a period of 13 minutes. This allowed participants to focus on the content and understand the essential points regarding schizophrenia. Second, we limited enrolment to parents of adolescents. Because the first symptoms of schizophrenia typically appear in adolescence [19,20], implementation of such a program for parents would be ideal for early detection and subsequent intervention for the disorder.

### Knowledge of schizophrenia

We attempted to measure participants' basic knowledge of schizophrenia. On the "before" questionnaire, the rate of correct answers for this section was 77%, indicating that the parents of junior and senior high school students were already knowledgeable about schizophrenia before viewing the education program. The education program resulted in a small improvement in basic knowledge, an increase of 3% (*P *< 0.001). Although the methods, participants, and results for education programs differ, many previous studies have shown that basic knowledge of schizophrenia is improved by such programs. Comptom et al. showed that a 40-hour training program for a crisis intervention team of 159 police officers in Atlanta resulted in a relative increase of 16%, from an average of 6.4 to 7.4 points, on a survey of general knowledge regarding schizophrenia [21]. Furthermore, a Japanese study suggested that some education programs for workers significantly improved knowledge of the effectiveness of drugs (before, 11.1%-61.3%; after, 33%-100%) [9]. In another study, Stuart [16] created a 20-minute video viewed by 571 Canadian high school students. The video described the signs and symptoms of schizophrenia, using a dramatization of a student worried about a friend's behaviour. Basic knowledge of schizophrenia has also been evaluated by several program sites in the World Psychiatric Association's global anti-stigma program. At baseline, 47.7% (*n *= 114) of students scored 80% or higher on items related to basic knowledge. This improved to 78.8% (*n *= 260) of students on the post-test (*P *< 0.001). As in other studies, enrolment of subjects in the present study was limited. In previous studies, family advocates and mental health consumer groups participated in the education program. In addition, those studies used the real-life experiences of individuals with schizophrenia to illustrate the educational concepts, a method that differs from the one used in the present study.

### Discrimination of schizophrenia symptoms

We also attempted to assess knowledge of the symptoms of schizophrenia, (i.e. the ability to differentiate schizophrenia symptoms from those of other disorders). In the present study, the rates of correct answers for items on prodromal symptoms and symptoms of schizophrenia were 30% and 47%, respectively, on the "before" questionnaire. These rates are not high, indicating that Japanese parents of adolescents are unable to discriminate the symptoms of schizophrenia from those of other disorders. Most individuals who develop schizophrenia experience a prodromal stage. In this phase before the initial psychotic episode, many symptoms may be present, including depressed mood, anxiety, irritability, changes in volition, cognitive changes, physical symptoms, behavioural changes, impaired tolerance to normal stress, and attenuated psychotic symptoms. However, common early prodromal symptoms are generally non-specific [4,7]. Parents of junior and senior high school students must be made aware of both schizophrenia symptoms and prodromal symptoms. Our findings were similar to those of an Italian study [22] which found that although most lay respondents were able to recognize the presence of a pathological condition in a vignette and acknowledge that it required psychiatric treatment, only 21% of them identified the condition as schizophrenia. Inadequate knowledge of schizophrenia, namely, limited ability to discriminate its symptoms, may lead to delayed detection of schizophrenia and prolonged duration of untreated psychosis [6]. After viewing the present education program, there were relative increases of 31% and 14% in the rates of correct answers for the items regarding discrimination of prodromal symptoms and the symptoms of schizophrenia, respectively, on the "after" questionnaire. These findings suggest that education programs can improve the ability to discriminate between schizophrenia and other disorders, and web-based presentations using a limited number of slides in a short time can be effective.

Finally, the present education program might be useful in testing parents of junior and senior high school students from other countries. Findings from such studies would aid in revising the questionnaire.

## Conclusions

Our web-based education program was useful in helping parents to acquire a basic knowledge of schizophrenia and discriminate correctly the symptoms of this disorder. The education program can be completed in only 13 minutes. Implementation of our program for parents could assist in early detection and subsequent intervention for schizophrenia.

## Declaration of competing interests

The authors declare that they have no competing interests.

## Authors' contributions

HY and KA designed the study and drafted the manuscript. YW, HK, and JC made significant contributions to the content of the paper and were responsible for the final editorial revision. All authors read and approved the final manuscript.

## Appendix I: Discrimination of schizophrenia symptoms

1. The person is not able to take a train because of a racing heart, feeling smothered, dizziness, and/or cold sweats.

2. During the third session, the patient becomes frightened, locks the door, and reports being chased by a spy.

3. The person feels weak, has a fever and mouth sores, as well as a rash over their entire body.

4. I think I might feel like my mind is 'playing tricks' on me.

5. The levels of the person's shoulders are different.

6. The person will not talk to their parents, takes a defiant attitude toward them, and seems to be hiding something.

7. I believe that I have special natural or supernatural gifts beyond my talents and natural strengths.

8. The patient has refused to eat for several weeks saying that the food may be poisoned.

9. The person has complained for quite a long time, saying that the TV or newspaper is reporting something bad about them.

10. The person is not able to feel enthusiasm because of depression and feels that life is hopeless. Also, the person is not able to sleep and has no appetite.

11. The person is not able to make decisions without help and is intimidated by parents or teachers.

12. The person persists in excessive dieting to decrease or maintain body weight.

13. I have thought that it might be possible that other people can read my mind, or that I can read other's minds.

14. The person suffers repeated abdominal pain or diarrhoea both while commuting by train to school and at school.

15. During summer vacation, the person could be heard arguing, although no one else was present.

16. The person has started coming home late and disobeying their parents.

17. I think that I may get confused at times whether something I experience or perceive may be real or may be just part of my imagination or dreams.

18. The person has started eating much more meat than vegetables.

19. There is no validity in the above-mentioned items.

## Appendix II: Reliability of basic knowledge survey

We performed a retest of basic knowledge of schizophrenia in the same manner as the first survey. We used the same questionnaire for a different sample of 735 individuals who had not been exposed to our web-based education program. Demographic characteristics of the respondents and the rates of correct answers are shown in Tables 5 and 6.

**Table 5:** Demographic characteristics of respondents (*n *= 735; second survey)

Characteristic	***n***	%
**Age (years)**		
30-39	41	5.6
40-49	541	73.6
50-59	152	20.7
60-69	1	0.1
**Gender**		
Male	389	52.9
Female	346	47.1
**Education**		
Junior high school	7	1.0
High school	217	29.5
Vocational school	97	13.2
Junior college	83	11.3
University	298	40.5
Graduate school	32	4.4
Others	1	0.1
**Family structure**		
2 parents	568	77.3
1 parent	27	3.7
3 generations	129	17.6
Others	11	1.5
**Family income (US dollars)**		
< 11,000	14	1.9
11,000-32,000	58	7.9
32,000-53,000	167	22.7
53,000-110,000	386	52.5
> 110,000	110	15.0
**Proximity to person with schizophrenia**		
Yes	32	4.4
No	703	95.6
**Occupation**		
Agriculture, forestry and fisheries	3	0.4
Production labour service	175	23.8
Transportation and communication	38	5.2
Sales and marketing	94	12.8
Service industry	108	14.7
Professionals	121	16.5
Others	37	5.0
Unemployed	159	21.6
**Participation in welfare activities for people with mental illness**		
Yes	49	6.7
No	686	93.3

**Table 6:** Rates (%) of correct answers for 14 items on basic knowledge of schizophrenia (second survey)

Item	Correct answer	"before" (*n *= 735)	"after" (*n *= 628)	*P*
1. The incidence of schizophrenia is 1 out of 100 individuals.	True	9.7	29.9	< 0.001
2. Junior and senior high school students do not develop schizophrenia.	False	99.0	97.5	NS
3. Schizophrenia is not a mental illness.	False	92.7	91.6	NS
4. Differences in child-rearing methods are not responsible for the onset of the illness.	True	21.0	33.3	< 0.001
5. A person with a mild disposition will not necessarily develop schizophrenia.	True	24.4	28.7	< 0.05
6. Schizophrenia has been proven to be a genetic disease.	False	98.0	98.4	NS
7. A person with schizophrenia may have little will power.	False	97.7	97.3	NS
8. There is no effective medication for schizophrenia.	False	95.0	96.5	NS
9. It is not possible to recover from schizophrenia, even with treatment.	False	98.1	98.4	NS
10. Auditory hallucinations and delusions are not symptoms of schizophrenia.	False	97.4	99.2	< 0.05
11. Poor concentration and fatigue are not symptoms of schizophrenia.	False	98.6	98.4	NS
12. Social withdrawal is not a symptom of schizophrenia.	False	93.3	93.5	NS
13. The appearance and duration of symptoms can vary among individuals.	True	61.0	58.3	NS
14. All the above items are incorrect.	False	81.2	83.8	NS
Total score		76.2	78.9	< 0.001

The distributions of demographic characteristics are almost identical to those in Table 1 (first survey). In addition, the rates of correct answers for the 14 items are similar to those in Table 2, although there are some significant differences.

## Appendix III

**Table 7:** Rates (%) of correct answers for 5 items on discrimination between disorders (second survey; *n *= 735)

Item	"before" *n *= 735	"after" *n *= 628	P*
Discrimination of schizophrenia symptoms	44.8%	53.3%	< 0.05
Discrimination of prodromal symptoms	26.7%	36.6%	< 0.001
Discrimination of other mental illnesses	69.6%	67.9%	NS
Discrimination of physical disorders	94.5%	91.5%	< 0.05
Discrimination of individuals who display unusual or antisocial behaviour	94.0%	91.5%	NS

*McNemar's test

## Pre-publication history

The pre-publication history for this paper can be accessed here:

http://www.biomedcentral.com/1471-2458/11/323/prepub
